# The Effect of Interbody Cage Parameters on the Rate of Subsidence in Single-Level Anterior Cervical Discectomy and Fusion (ACDF): A Retrospective Analysis of 98 Patients

**DOI:** 10.7759/cureus.50386

**Published:** 2023-12-12

**Authors:** Aleeza Safdar, Benjamin Headley, Marcus Rommelman, Ahmad Haseeb, Rouzbeh Motiei-Langroudi

**Affiliations:** 1 Neurosurgery, University of Kentucky, Lexington, USA; 2 Physical Medicine and Rehabilitation, University of Kentucky, Lexington, USA; 3 Radiology, Lehigh Valley Hospital, Allentown, USA; 4 College of Medicine, University of Kentucky, Lexington, USA

**Keywords:** interbody cage fusion, cervical spine stenosis, cervical disc herniation, anterior plating system, subsidence, cage, interbody, acdf, anterior cervical discectomy fusion

## Abstract

Introduction: Subsidence is a relatively common consequence after anterior cervical discectomy and fusion (ACDF) surgery. This study aimed to identify the effect of radiological and non-radiological risk factors on subsidence after a single-level ACDF surgery with cage and plate.

Methods: This is a retrospective cohort study of patients who underwent ACDF for radiculopathy or myelopathy at an academic center, University of Kentucky Albert Chandler Hospital, Lexington, Kentucky, United States, between January 2010 and January 2020. Subsidence was defined as the sinking of the interbody cage into the vertebral body at either the superior end plate (SEP) or inferior end plate (IEP) at the ACDF level and was measured manually on lateral standing x-ray. The numerical amount of subsidence was measured in millimeters as the sum of subsidence in the SEP and IEP and was further categorized into subsidence2 and subsidence3 (i.e., presence of subsidence > 2 mm and subsidence > 3 mm, respectively). Multivariate regression analysis was used to assess the effect of variables such as age, gender, body mass index (BMI), tobacco use, follow-up length, cage type, anterior cage height, posterior cage height, anterior cage height ratio, posterior cage height ratio, cage position, cage-end plate interface and cervical alignment on outcomes such as subsidence, subsidence2, and subsidence3.

Results: A total of 98 patients were included, of which 46 (47.1%) were male. The mean age of the population was 47.6±8.4 years. Fifty-one patients (52%) experienced subsidence more than 3 mm. Anterior disc height ratio (ADHR) was calculated by dividing the anterior cage height by the anterior disc height (pmADH). The posterior disc height ratio (PDHR) was calculated by dividing the posterior cage height by the posterior disc height (pmPDH). There was no significant correlation between ADHR and PDHR with subsidence, (p=0.93 and 0.56, respectively). Gender, age, BMI, and smoking status did not affect subsidence either. Cage type significantly affected subsidence with a higher subsidence rate in VG2 cages compared to Bengal cages (p=0.05).

Conclusion: This study showed that in patients undergoing single-level ACDF with cage and plate, cage size and in particular cage height (if adjusted for individual patients) did not affect subsidence. Other factors such as cage-endplate interface, cage depth in interbody space, and cervical alignment did not significantly affect subsidence either. This might be attributable to the use of an anterior plating system that conducts the force and reduces the stress on the graft-bone interface.

## Introduction

Anterior cervical discectomy and fusion (ACDF) is a widely performed surgical treatment for radiculopathy or myelopathy in degenerative or traumatic cervical disease for patients who are refractory to conservative management [1,2]. Fusion can be achieved by using intervertebral cervical interbody cages with or without anterior plates and screws [3].

Subsidence, defined as the sinking of the interbody cage into the vertebral body, is a relatively common consequence [4]. According to a recent systematic review, cage subsidence has an overall incidence of around 21% after ACDF [5]. Cage subsidence can lead to nonunion, loss of segmental lordosis, kyphotic malalignment of the cervical spine, and recurrent stenosis [5]. Factors influencing subsidence are age, cervical alignment, using (or not using) plates, site-specific bone mineral density, location of cage placement, and cage material [6-10].

There is inconclusive data on the relation of cage height and subsidence after ACDF with cage and plate. While some studies report that the greater the cage height, the greater the risk of cage subsidence [11,12], others have reported that cage height is not associated with cage subsidence [8]. However, many of these studies have investigated ACDF using stand-alone cages (i.e., without the use of a plate), as plates have been shown to reduce the rate of subsidence [13].

The purpose of our paper is to evaluate the effect of demographic, radiologic, and cage and endplate-related parameters on cage subsidence in patients undergoing single-level ACDF with the use of interbody cage and plate. The primary outcome of interest was the effect of cage size on subsidence (if adjusted for each patient by calculating the disc height ratio).

## Materials and methods

This is a retrospective cohort study of patients who underwent ACDF for radiculopathy or myelopathy at an academic center, University of Kentucky Albert Chandler Hospital, Lexington, Kentucky, United States, between January 2010 and January 2020. Eligible patients were identified through medical and billing records. The study was approved by the University of Kentucky Institutional Review Board (approval number: 81592).

Inclusion and exclusion criteria

Adult patients (18-90 years old) with degenerative pathology such as cervical disc herniation and cervical spine stenosis with a single-level surgery between C3-C7 and use of interbody cage plus anterior plating system, who had a pre-operative MRI plus a pre-operative and post-operative standing follow-up cervical x-ray were included. Patients who had a history of any previous cervical spine surgery, non-degenerative pathologies such as infection, tumor, or trauma, and surgery on levels C2-C3 and C7-T1 were excluded. Patients who did not have the necessary follow-up imaging such as lack of a standing follow-up x-ray were also excluded.

Research variables

Demographic and Surgical Variables

**Figure 1:**
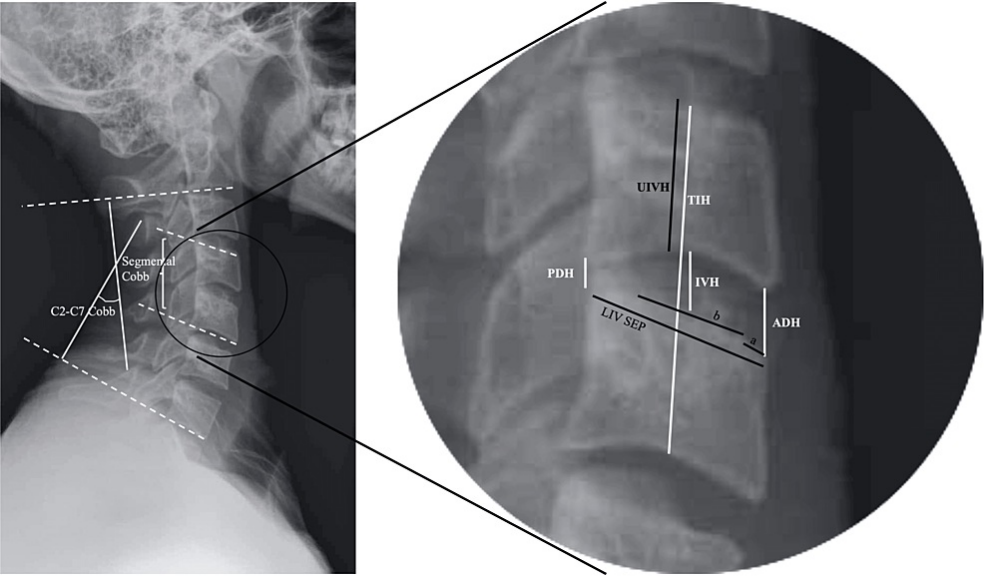
Schematic illustration of the pre-operative and post-operative radiological measurements on x-ray. Cage depth within interbody space is measured as the ratio of a/LIV SEP. Cage-endplate interface is measured as b/LIV SEP. ADH: anterior disc height; IVH: intervertebral height; PDH: posterior disc height; TIH: total intervertebral height; UIVH: upper instrumented vertebra height; LIV SEP: lower instrumented vertebra superior endplate Image credits: Rouzbeh Motiei-Langroudi

The variables studied were age, sex, body mass index (BMI), smoking status, indication for surgery, date of surgery, level of ACDF, follow-up length, cage type, cage size (actual anterior height, posterior height, and width provided by the manufacturer), and cage position within interbody space. A representative illustration of the radiological measurements is shown in Figure 1.

Imaging Parameters

The following imaging parameters were measured and reviewed on the pre-operative MRI for the disc that underwent ACDF: (i) anterior disc height (pmADH), i.e. height at the anterior aspect of the disc; (ii) posterior disc height (pmPDH), i.e. height at the posterior aspect of the disc; (iii) upper instrumented vertebra height (pmUIVH), i.e. height of the vertebral body of the UIV (in the middle).

The following imaging parameters were measured and reviewed on the pre-operative standing x-ray for the disc that underwent ACDF: (i) anterior disc height (pxADH); (ii) intervertebral height (pxIVH); (iii) posterior disc height (pxPDH); (iv) total intervertebral height (pxTIH); (v) upper instrumented vertebra height (pxUIVH); (vi) C2-C7 Cobb angle on the lateral standing x-ray (pCC); (vii) segmental Cobb angle (UIV’s superior end plate to lower instrumented vertebra, LIV’s inferior endplate) on the lateral standing x-ray (pSC).

The following imaging parameters were measured and reviewed on the post-operative follow-up lateral standing x-ray: (i) anterior disc height (fxADH); (ii) intervertebral height (fxIVH); (iii) posterior disc height (fxPDH); (iv) total intervertebral height (fxTIH); (v) upper instrumented vertebra height (fxUIVH); (vi) C2-C7 Cobb angle (fCC); (vii) segmental Cobb angle (fSC); (viii) cage depth within interbody space (ratio of the distance of anterior margin of the cage to the anterior margin of LIV to the length of superior endplate of LIV; (ix) cage-endplate interface (ratio of anterior-posterior length of the cage to the length of superior endplate of LIV), and presence of subsidence. C2-C7 Cobb angle change (CCC) was calculated as fCC − pCC.

To adjust for the individual variability in disc height between patients, disc height ratios were calculated. Anterior disc height ratio (ADHR) was calculated by dividing the anterior cage height by the anterior disc height (pmADH). The posterior disc height ratio (PDHR) was calculated by dividing the posterior cage height by the posterior disc height (pmPDH).

Subsidence

**Figure 2:**
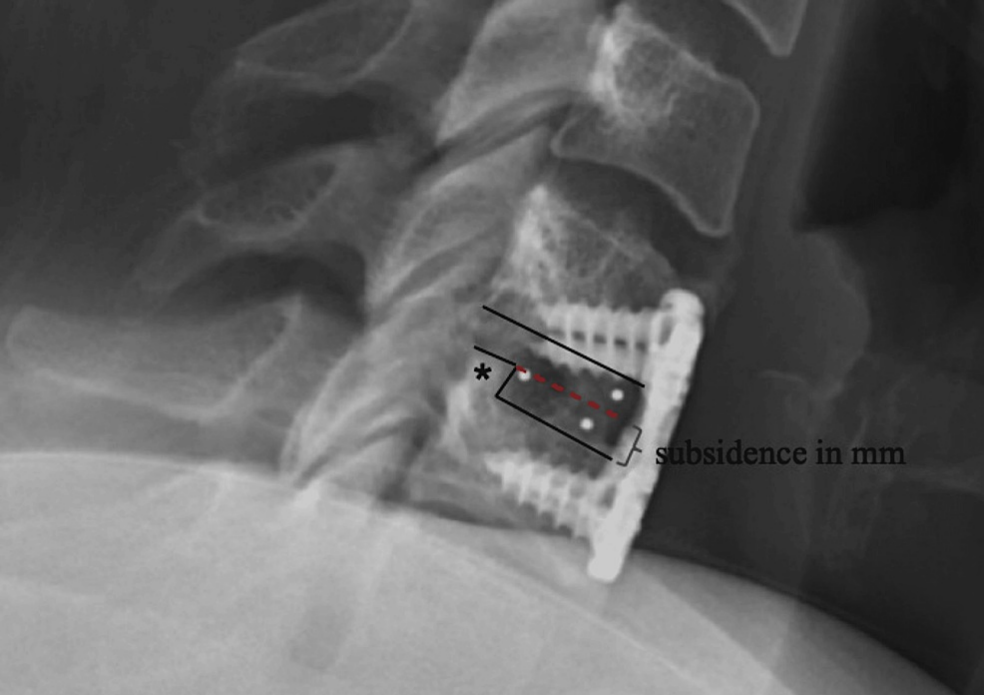
Measurement of subsidence. At the ACDF level, the outlines of superior endplate (SEP) of the lower instrumented vertebra and inferior endplate (IEP) of the upper instrumented vertebra were marked first (solid black lines). In the presence of subsidence, a leveling can be observed in SEP, IEP, or both (asterisk). Then, an imaginary intact endplate (as if no subsidence had happened) would be marked (dashed red line). The distance between the two lines was measured to represent subsidence (gray bracket). Image credits: Rouzbeh Motiei-Langroudi

Subsidence was defined as the sinking of the cage into the anterior and/or posterior border of the superior endplate (SEP) or inferior end plate (IEP) at the ACDF level [4]. The numerical amount of subsidence was measured in millimeters as the sum of subsidence in SEP and IEP and further categorized into subsidence2 and subsidence3 (i.e., presence of subsidence > 2 mm and subsidence > 3 mm, respectively). An illustration of the measurements is shown in Figure 2.

Subsidence was recorded and measured manually on lateral standing follow-up x-rays by three independent examiners. The examiners were not blinded to the outcome of the study. To verify subsidence values measured on x-ray, the following mathematical formula was used to calculate subsidence based on the vertebral body and disc heights before and after cage insertion:

Subsidence-mathematical = (pxTIH − pxIVH) − (fxTIH − fxIVH)

In the absence of subsidence, pxTIH − pxIVH should be equal to fxTIH − fxIVH, as both would measure for the sum of the heights of the upper and lower instrumented vertebrates. However, in the presence of subsidence, a positive value should be yielded. To adjust for the magnification observed on pre-operative x-ray (pxMagnification), the measurements were compared to the measurements taken from MRI (pxUIVH/pmUIVH), as MRI lacks a magnification error. For the post-operative x-ray (fxMagnification), we used the comparison between measured and actual disc height on the post-operative x-ray (fxADH/anterior cage height). We then used the same formula as above to calculate magnification-controlled subsidence:

Subsidence-mathematical2 = ((pxTIH − pxIVH) / pxMagnification) − ((fxTIH − fxIVH) / fxMagnification).

Statistical analysis

All data were analyzed with IBM SPSS Statistics for Windows, Version 28.0 (Released 2021; IBM Corp., Armonk, New York, United States). Chi-square and independent t-test tests were used to analyze univariate qualitative and quantitative data, respectively. To predict the effect of independent variables on outcomes, multivariate regression analysis was used with variables that had a p-value less than 0.1 on univariate analysis (chi-square and t-tests). Multivariate binomial regression analysis was used to assess the effect of independent variables on outcomes such as subsidence, subsidence2, and subsidence3. P-values < 0.05 were considered statistically significant.

## Results

A total of 1174 patients were identified through medical and billing records and were initially reviewed. Of these, 98 patients were finally included in the current study after meeting the inclusion criteria. The age range was 28-71 years. The mean follow-up (±SD) was 20.6 ± 25.3 months. Demographic characteristics of the study population and statistics for subsidence are shown in Table 1.

**Table 1:** Demographics of the study population and statistics for subsidence. Each cell represents mean ± SD for quantitative variables and number (%) for qualitative variables. Subsidence: any amount of subsidence; subsidence2: subsidence > 2 mm; subsidence3: subsidence > 3 mm.

Variables	Values
Age (years), Mean ± SD	47.6±8.4
Body mass index BMI (kg/m^2^), Mean ± SD	31.6±8.4
Gender, n (%)	
Male	46 (47.1%)
Female	52 (52.9%)
Smoking status, n (%)	
Smoker	44 (45.2%)
Non-smoker	43 (44%)
Past-smoker	11 (10.8%)
Subsidence, n (%)	
Subsidence	84 (85.7%)
Subsidence2	65 (66.3%)
Subsidence3	51 (52%)

Age, BMI, gender, the level of surgery, pmADH, pmUIVH, pmPDH, pxADH, pxIVH, pxPDH, pCC, pSC, fxPDH, cage depth within interbody space, cage-endplate interface and CCC did not significantly influence the occurrence of subsidence. However, cage type significantly affected subsidence (higher subsidence rate in VG2 cages compared to Bengal cages; p=0.05). Multivariate regression analysis showed none of the variables in the model (age, gender, BMI, tobacco use, follow-up length, cage type, anterior cage height, posterior cage height, anterior cage height ratio, posterior cage height ratio, cage depth, and cage-endplate interface) significantly predicted subsidence (p = 0.91, 0.63, 0.55, 0.71, 0.07, 0.40, 0.29, 0.48, 0.86, 0.66, 0.45, 0.95, respectively). For subsidence2, only follow-up length and anterior cage height significantly predicted the outcome (p = 0.04 and 0.01, respectively), while other variables did not influence the outcome (p>0.2 for all). For subsidence3, again only follow-up length and anterior cage height significantly predicted the outcome (p = 0.002 and 0.01, respectively), while other variables did not influence the outcome (p>0.3 for all). The effect of radiologic and non-radiologic parameters on subsidence is demonstrated in Table 2. 

**Table 2:** Effect of radiologic and non-radiologic parameters on subsidence. Each cell represents mean ± SD for quantitative variables and number (%) for qualitative variables. Subsidence: any amount of subsidence; subsidence2: subsidence > 2 mm; subsidence3: subsidence > 3 mm. *denotes statistical significance; #statistical analysis not performed since all cases had the same value. ACDF: anterior cervical discectomy and fusion; CCC: C2-C7 cobb angle change (post-operative measure – pre-operative measure); fxADH: anterior disc height (ADH) on the follow-up x-ray; fxIVH: intervertebral height (IVH) on the follow-up x-ray; fxPDH: posterior disc height (PDH) on the follow-up x-ray; fxTIH: total intervertebral height (TIH) on the follow-up x-ray; fxUIVH: upper instrumented vertebra height (UIVH) on the follow-up x-ray; pCC: C2-C7 cobb angle on the pre-operative x-ray; pmADH: ADH on the pre-operative MRI; pmPDH: PDH on the pre-operative MRI; pmUIVH: UIVH on the pre-operative MRI; pSC: segmental cobb angle (upper instrumented vertebra’s superior end plate to lower instrumented vertebra’s inferior end plate) on the pre-operative x-ray (pSC); pxADH: ADH on the pre-operative x-ray; pxIVH: intervertebral height (IVH) on the pre-operative x-ray; pxPDH: PDH on the pre-operative x-ray; pxTIH: total intervertebral height (TIH) on the pre-operative x-ray; pxUIVH: UIVH on the pre-operative x-ray.

Variables	Subsidence			Subsidence2			Subsidence3		
	Yes	No	p-value	Yes	No	p-value	Yes	No	p-value
Age (years)	47.50±8.58	48.71±8.26	0.62	48.35±8.38	46.33±8.73	0.27	47.59±8.14	47.77±8.98	0.92
BMI (kg/m^2^)	31.37±8.29	33.51±10.24	0.39	31.14±7.78	32.71±9.97	0.40	31.07±8.42	32.32±8.77	0.48
Gender			0.16			0.08			0.30
Male	42 (91.1%)	4 (8.9%)		35 (75.6%)	11 (24.4%)		27 (57.8%)	19 (42.2%)	
Female	42 (81.1%)	10 (18.9%)		30 (58.5%)	22 (41.5%)		25 (47.2%)	27 (52.8%)	
Smoking status			0.92			0.31			0.20
Smoker	37 (83.3%)	7 (16.7%)		31 (69.4%)	13 (30.6%)		24 (55.6%)	20 (44.4%)	
Non-smoker	36 (83.3%)	7 (16.7%)		24 (55.6%)	19 (44.4%)		17 (38.9%)	26 (61.1%)	
Past-smoker	9 (77.9%)	2 (22.2%)		9 (77.9%)	2 (22.2%)		7 (66.7%)	4 (33.3%)	
Imaging parameters									
pxADH	4.40±1.39	4.17±1.88	0.58	4.45±1.40	4.21±1.59	0.45	4.55±1.24	4.17±1.66	0.20
pxIVH	5.57±1.73	5.62±1.96	0.92	5.60±1.87	5.53±1.53	0.87	5.51±1.36	5.64±2.12	0.71
pxPDH	4.10±1.22	3.84±1.45	0.48	4.07±1.18	4.03±1.39	0.88	4.12±1.05	3.99±1.44	0.60
pxTIH	28.57±4.98	28.43±7.02	0.93	28.63±5.16	28.38±5.57	0.83	28.50±4.96	28.60±5.65	0.92
pxUIVH	11.88±3.00	10.84±1.22	0.21	12.02±3.26	11.17±1.62	0.16	11.64±2.70	11.83±3.01	0.74
pmUIVH	10.67±1.43	10.62±1.21	0.89	10.68±1.50	10.63±1.17	0.87	10.63±1.60	10.69±1.14	0.83
pmADH	4.20±1.30	4.44±1.51	0.54	4.28±1.30	4.16±1.38	0.69	4.28±1.37	4.19±1.29	0.74
pmPDH	3.66±1.04	3.70±1.51	0.91	3.64±1.01	3.72±1.29	0.75	3.64±1.01	3.70±1.22	0.81
pCC	9.65±8.81	11.50±7.70	0.46	9.60±8.81	10.55±8.43	0.61	8.92±9.06	11.00±8.15	0.24
pSC	3.64±6.67	6.43±4.01	0.13	3.69±7.11	4.73±4.81	0.45	3.76±7.26	4.34±5.42	0.66
CCC	-0.56±8.54	1.39±8.83	0.43	-0.49±7.23	0.14±10.77	0.74	0.51±6.90	-1.10±10.02	0.36
fxADH	6.67±2.22	5.42±2.31	0.06	6.49±2.41	6.49±1.99	0.10	6.45±2.52	6.53±1.97	0.87
fxIVH	5.51±1.93	4.43±1.68	0.05*	5.41±2.03	5.25±1.72	0.70	5.35±2.14	5.37±1.68	0.98
fxPDH	3.89±1.38	3.24±1.86	0.13	3.76±1.32	3.88±1.74	0.69	3.61±1.34	4.00±1.57	0.18
fxTIH	34.07±4.46	33.14±3.79	0.46	34.15±4.62	33.52±3.85	0.50	34.42±4.66	33.42±3.10	0.26
fxUIVH	14.00±2.48	14.61±1.93	0.39	14.05±2.58	14.15±2.06	0.84	14.11±2.79	14.06±1.94	0.92
Cage parameters									
Anterior cage height	7.32±0.82	7.02±0.88	0.24	7.37±0.80	7.10±0.88	0.14	7.40±0.86	7.15±0.78	0.15
Posterior cage height	6.09±0.81	5.80±0.85	0.25	6.13±0.79	5.90±0.87	0.19	6.15±0.85	5.94±0.77	0.21
Cage AP diameter^#^	12.00±0.00	12.00±0.00		12.00±0.00	12.00±0.00		12.00±0.00	12.00±0.00	
Cage width^#^	14.50±0.00	14.5±0.00		14.50±0.00	14.50±0.00		14.50±0.00	14.50±0.00	
ADHR	1.97±0.99	1.94±1.45	0.93	1.97±1.07	1.96±1.04	0.98	2.01±1.16	1.92±0.92	0.69
PDHR	1.76±0.52	1.86±0.77	0.56	1.78±0.53	1.76±0.61	0.87	1.75±0.45	1.79±0.67	0.73
Cage type			0.05*			0.08			0.04*
Bengal	86.70	13.30		63.9	36.1		50.6	49.4	
VG2C	91.70	8.30		91.7	8.3		75.0	25.0	
ACDF level			0.07			0.08			0.53
Cage depth within interbody space (%)	13.57±7.58	12.24±6.43	0.55	12.92±6.91	14.30±8.36	0.41	13.46±7.57	13.29±7.31	0.92
Cage-endplate interface (%)	51.31±16.94	48.67±7.16	0.58	50.84±18.71	51.08±7.64	0.95	51.78±20.84	50.02±8.10	0.61

Influence of cage size and location on subsidence

Anterior cage height, posterior cage height, cage width, ADHR, and PDHR did not affect subsidence as demonstrated in Table 2. Pearson correlation showed that there was a significant correlation between the numerical amount of subsidence and anterior cage height (r = 0.23; p=0.03), but no significant correlation was noticed between measured subsidence, subsidence-mathematical, and subsidence-mathematical2 with ADHR (r = 0.07, -0.09, -0.18; p = 0.51, 0.40, 0.08, respectively) and PDHR (r = -0.01, -0.04, -0.19; p = 0.93, 0.7, 0.07; respectively). No statistical analysis was performed for cage width and anterior-posterior diameter since all cases had the same value. Moreover, cage depth within interbody space and cage-endplate interface did not affect subsidence as demonstrated in Table 2. No significant correlation was noticed between measured subsidence, subsidence-mathematical, and subsidence-mathematical2 with cage depth (r = -0.02, 0.07, 0.06; p = 0.85, 0.52, 0.59, respectively) and cage-endplate interface (r = -0.04, 0.02, 0.04; p = 0.69, 0.88, 0.71; respectively).

## Discussion

It has been shown in previous studies that there is a significant association between cage height and subsidence for stand-alone cages, but there is inconclusive data on the ideal cage height to avoid subsidence when ACDF is performed with cage and plate [11]. Although initially, we observed a significant linear correlation between numerical amount of subsidence and anterior cage height, we believe this is a confounding factor because when the cage height was adjusted for each individual patient by calculating the anterior and posterior disc height ratio (ADHR and PDHR), no significant correlation was seen with the numerical amount of subsidence, subsidence2 or subsidence3. The biggest strength of this study is checking the ratio of cage size to disc height. Thats why we believe individual numbers are not truly reflective. For a patient with a 5 mm disc height putting a 7 mm cage will significantly stretch the disc space while putting a 7 mm cage in a patient who already has a 7 mm disc height doesn't do any stretching. As such, pure numbers are not reflective of any change here while the ratios truly matter. In other words, cage size should be adjusted relative to individual patient disc height and tailored according to individual patient. Similar to our results, Truumees et al. reported in their study that pre-operative disc height was not a significant predictor of compression of the graft [12]. We found a significant correlation between fxIVH and subsidence in the current study, but again, this is a confounding factor and has no clinical significance because fxIVH must be corrected for each individual patient like the concept discussed for cage size. Moreover, the significance was not observed in multivariate regression analysis.

No significant correlation was found in our study between pCC, pSC, or CCC with subsidence. It has been shown previously that pre-operative cervical alignment has a significant correlation with subsidence in patients with ACDF without the use of plates, but no significant correlation in patients with the use of plates [10]. All patients in our study had cage placement with the use of plates and so the results are consistent with other reports showing that cervical alignment does not have a significant correlation with subsidence in patients with ACDF with cage and plate. It is important to note that patients who undergo ACDF with a cage-only technique are reported to have increased rates of subsidence, decreased post-operative disc height, and less restoration of cervical lordosis in comparison to patients who undergo ACDF with a cage-plate technique [4]. The biomechanical explanation for this is that with anterior plating, the mechanical force over the instruments is shared through the anterior plate and screws (added to the cage) and as such, there is less contact stress at the cage-endplate interface, reducing subsidence and hardware failure in general. In other words, the anterior plate augments stabilization of the fusion construct by sharing the load and hence altering the biomechanics of the fused spine such that the risk of subsidence is lower with the use of plates compared to stand-alone cages [13].

Cage material is reported to be a risk factor for subsidence in prior literature; polyetheretherketone (PEEK) vs. titanium cages have significantly different subsidence levels for cage height>5.5 mm but not <5.5 mm [10]. In the present study, there was a significant difference in subsidence between Bengal vs. VG2 cages. It might be due to the ability to use autograft within Bengal cages, but this merits a more focused study. Moreover, the whole series included mostly two cage types only, so we are unable to make a broader and more in-depth analysis of the effect of cage type on subsidence other than these two cage types. Other risk factors for subsidence include a smaller anteroposterior cage diameter, and a smaller cage surface area resulting in reduced end-plate coverage that increases the stress on the endplate [14]. In our study, the cage-endplate interface as measured by the ratio of cage anterior-posterior length to superior endplate length did not affect subsidence. The anterior portion of the endplate is richer in cortical bone, and hence cage position towards the posterior aspect of the vertebral body is also reported to increase subsidence in prior literature [14]. However, cage depth in interbody space was not significantly associated with subsidence in the present analysis. Again, the results in the patients of the current study might be attributable to the use of an anterior plating system that enhances stability and reduces the stress on the implant-bone interface as the loading pressure that is directly delivered to the cage/end plate interface can be shared by the anterior metal plating [15]. This is the first study to investigate the effect of cage size in ACDF with cage and plate, as previous reports are for stand-alone cages.

Prior literature has different definitions of subsidence: Barsa et al. defined subsidence as > 2 mm reduction in segmental height due to implant migration into the adjacent end plates [8], whereas Gercek et al. defined subsidence as a change in implant migration of ≥ 3 mm [16]. Hence, we have used different measuring and statistical techniques. Subsidence was divided into categorical vs. numerical and then numerical subsidence was further categorized into subsidence2, and subsidence3. To control for the operant error, we calculated subsidence in an indirect way (i.e., the formula for subsidence-mathematical). We used pre-operative MRI scans to measure pmADH and pmPDH to make the readings more precise and eliminate magnification variation in readings taken from plain x-rays. Radiographic measurements were done by three independent examiners to eliminate inter-observer bias and increase accuracy. In prior literature, subsidence has been reported to range from 13.2% to 62.5% of cases [7,8,16-18]. In the present study, 52% of the patients experienced subsidence3, i.e., more than 3 mm.

Our study is limited by its retrospective nature. For our patient population, two cage types were used, so we are unable to make a broader and more in-depth analysis of the effects of cage type on subsidence other than the two cage types that have been discussed earlier. Bone mineral density and surgeon-related factors are commonly reported to affect rates of cage subsidence in literature; however, for this study, we did not have data available for these factors. The complex biomechanical distractive forces operating at the implant-bone-plate unit were not a focus of our study and have not been studied in this retrospective review. Future prospective studies including more patients are required to fully investigate the factors affecting cage subsidence including cage and endplate-related factors, specifically looking into optimal cage size for individual patients.

## Conclusions

This study showed that in patients undergoing single-level ACDF with cage and plate, cage parameters including cage size (in particular cage height, if adjusted for individual patient and disc height) did not affect subsidence. Other factors such as cage location within disc space, cage-endplate interface, and cervical alignment did not significantly affect subsidence either. This might be attributable to the use of anterior plating system that reduces the stress on the graft-bone interface. This allows surgeons to chose reasonably larger cages to help with indirect decompression of the central and foraminal spaces, without fear of hardware failure.
